# Effect of metformin on free testosterone level in male diabetic patients: A comparative study

**DOI:** 10.1097/MD.0000000000044989

**Published:** 2025-11-07

**Authors:** Md Aminul Islam, Md Azizul Haque, Abdul Mumit Sarkar, Md Siddiqur Rahman, Al Muksit Mohammad Taufiqur Rahman, Shamrose Begum

**Affiliations:** a Department of Medicine, Rajshahi Medical College, Rajshahi, Bangladesh.

**Keywords:** free testosterone level, male diabetic patients, metformin

## Abstract

When we consider the treatment of polycystic ovary syndrome (PCOS), one of our treatment options is metformin, which reduces free testosterone levels in PCOS patients. We were interested to know what happens to the free testosterone levels of male type 2 diabetes mellitus (T2DM) patients when we use metformin. The effect of metformin on testosterone level, fertility, and sexual activity in males has been issues of interest to researchers. This study was conducted to observe the effect of metformin on free testosterone levels in male T2DM patients. One hundred fifty male patients (mean age, 40–46 years*; SD, 1.2*), newly diagnosed with T2DM and drug-naïve, were enrolled. Initially, the baseline-free testosterone level was measured. Then, 75 of them were given metformin, and 75 of them were treated with other antidiabetics. After treatment for 30 days, the free testosterone level was measured again. In both groups, the mean value of free testosterone change in 1 month was calculated. Then, a comparison between the mean change in the 2 groups was done. Baseline-free testosterone of metformin (mean = 7.9, SD = 2.2) and non-metformin (mean = 8.1, SD = 1.9) groups were compared, and no significant difference was seen between them (*P* = .264). After 30 days of treatment, there was an increased level of free testosterone in both groups (mean metformin = 9.75, non-metformin = 10.77). But. free testosterone rise in non-metformin group(M = 2.63,SD = 1.07) was significantly higher (mean difference = .80,95% CI) than metformin group (M = 1.82,SD = .99,t = 4.76, *P* < .001). Metformin is inferior to other antidiabetic medications in raising free testosterone levels. Larger multicenter studies are further warranted.

## 1. Introduction

Metformin is a time-tested drug for the management of type 2 diabetes mellitus (T2DM) and is being used as a first-line oral hypoglycemic agent in both obese and non-obese patients. Although its central position in managing newly diagnosed T2DM has been challenged by SGLT-2 inhibitors due to their major cardiovascular events (MACE) risk-lowering effects, in clinical practice, it seems that it will keep its current position in the future.^[1]^ With time, scientists have established metformin for other indications; even, metformin is now being investigated for immunomodulatory effects.^[2]^

However, metformin is well known for its use in polycystic ovary syndrome(PCOS) and infertility. Studies showed modest androgen-lowering effects in PCOS, also in pregnant women.^[3]^ Although some studies showed no benefits of metformin in PCOS infertility and hyperandrogenism.^[4]^

An interrelationship between metformin, free testosterone level, skeletal muscle mass, and glycemic control will come. A study shows preserved muscle mass with testosterone therapy in the geriatric population, but there was no functional improvement or fatigability.^[5]^ Also, we searched for studies showing the relation of testosterone therapy and glycaemic control in T2DM, but we found no significant correlation,^[6]^ so it can be assumed that whatever the effect of metformin on testosterone level, it would not affect the glycaemic control.

Now, we are interested in exploring the effect of metformin on free testosterone levels in male T2DM patients. There are a few studies where researchers showed interest in the same issue and demonstrated the testosterone-lowering effect of metformin.^[7,8]^

Free testosterone level has ethnic variation. A study in the United States observed differences in various reproductive hormones and sperm count between White, Black, Asian, Hispanic, and non-Hispanic men and found significant differences. Specifically, Asian people had lower free testosterone levels than White people.^[9]^ Also, metformin response variation also occurs due to genetic variations.^[10]^ Due to ethnic and genetic variation, we need to conduct a study among Bangladeshi people separately. Moreover, we could not find any study conducted on this issue among people in Bangladesh. As metformin is used in many patients in Bangladesh, this study will be important for clinicians.

## 2. Patients and methods

### 2.1. Study design

It is a hospital-based prospective observational study conducted at both outpatient and inpatient settings of the Department of Medicine, Rajshahi Medical College Hospital, and Rajshahi Diabetic Association General Hospital, Bangladesh, from January 2024 to July 2024. The study was approved by an ethical review committee of Rajshahi Medical College, Bangladesh (Ref: RMC/ERC/2022/24[b]). Written informed consent was obtained from all persons participating in the study.

### 2.2. Sample size calculation:

Puposive sampling was done.

As we will compare 2 means, so,

Sample size:

${\;n=((z}_{\;\propto }{+\text{z}}_{\;\beta }{)}^{\;2}{\times (\sigma }_{\;1}^{\;2}{+\sigma }_{\;2}^{\;2}{))/(\mu }_{\;1}{-\mu }_{\;2}{)}^{\;2}$

${\left((1.96+.85\right)}^{\;2}{\times [(.09)}^{\;2}{+(.08)}^{\;2}{])/(.24-.20)}^{\;2}$

Considering this equation with reference to the previous study, we found the sample size to be 71.59, that is 72 with 80% power and 95%confidence level.^[8]^ Seventy 2 patients will form the case group, and another 72 will form the control group.

### 2.3. Patients and control groups

Newly diagnosed T2DM male patients between the ages of 18 and 60 years were treated with metformin and were enrolled as cases.

On the other hand, male patients with similar age ranges treated with other medications other than metformin were taken as controls.

Patients on a drug having antiandrogenic properties (finasteride, spironolactone, statin), or a previous history of hypogonadism were excluded from the study. Some studies show a significant reduction in testosterone in patients on statins.^[11,12]^

Both groups of patients were informed in detail about the steps of the study verbally and also through a written informed consent form written both in Bengali and English.

### 2.4. Measurement of serum-free testosterone

Serum-free testosterone was assessed twice, once at the time of enrollment and again 30 days later. 3 ml of venous blood was obtained from each patient and was left for 30 minutes for spontaneous clotting. Samples were then centrifuged at 3000 rpm for 5 minutes and were stored at −20 °C. Serum-free testosterone was measured by Abbott Architect i-1000-SR/VitrosECi System(J&J)/Advia Centaur CP(Siemens)Random Access Multibatch Immunoassay Analyzer.^[13]^

### 2.5. Statistical analysis

Data processing and analysis were done with SPSS (Statistical Package for Social Science), version 25. Numerical variables were presented as mean ± standard deviation, and categorical variables were described in absolute numbers and percentages. Continuous variables were compared using the Student *t*-test. The chi-square or Fisher exact test was applied in the appropriate field.

Baseline characteristics were compared between groups using an independent samples t-test for continuous variables (e.g., age) and a χ² test for categorical variables.

## 3. Results

**Figure 1.**
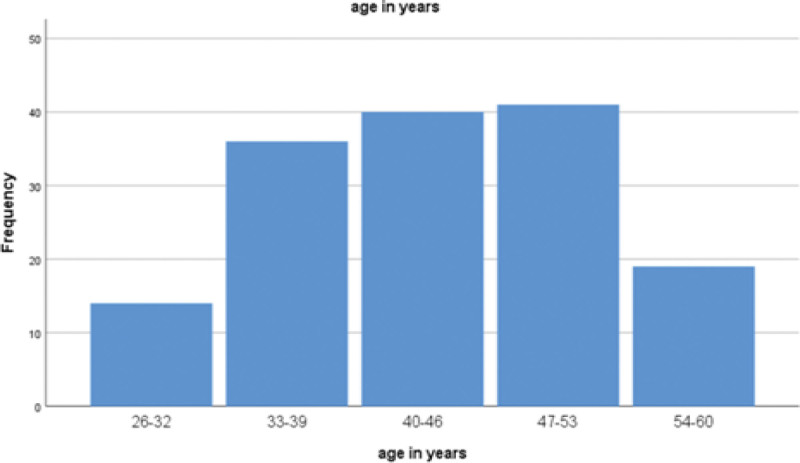
Age distribution of study participants among newly diagnosed diabetic male patients (bar chart).

General characteristics of study subjects: A total of 150 male newly diagnosed T2DM patients were enrolled. Age distribution is shown in Figure 1 through a bar chart.

The mean age of participants was 43.2 ± 6.4 years overall. There was no significant difference in mean age between the metformin group (43.0 ± 6.5 years) and the non-metformin group (43.4 ± 6.3 years, *P* = .237) (Table 1). Thus, the 2 groups were comparable in terms of age at baseline.

**Table 1:** Comparison between age of participants of metformin and non-metformin groups.

Group	Mean Age	SD	N	*P*-value
Metformin	43.0	6.5	75	.237
Non-metformin	43.4	6.3	75	
Total	43.2	6.4	150	

SD = standard deviation.

### 3.1. Baseline-free testosterone

Each newly diagnosed T2DM patient’s blood sample was taken to see the baseline-free testosterone level. No significant difference was observed in baseline-free testosterone levels between cases (mean = 7.9, SD = 2.2) and controls(mean = 8.1, SD = 1.9) (*P* = .264) (Table 2).

**Table 2:** Mean difference of baseline-free testosterone among metformin and non-metformin groups.

Baseline-free testosterone	Group	N	Mean	SD	*t*
	Metformin	75	7.9	2.2	.264*
	Non-Metformin	75	8.1	1.9	

SD = standard deviation.

* *P* < .05.

After 30 days of treatment, free testosterone level was measured in both the metformin and non-metformin groups. In both groups, there were increased levels of free testosterone (mean metformin = 9.75, non-metformin = 10.77), but the difference between them was statistically significant (*P* < .05) (Table 3).

**Table 3:** Independent sample *t*-test.

Treatment Group	N	Mean	SD	t	Sig (2 tailed)
Metformin	75	1.82	.99	4.758	000
Non-metformin	75	2.62	1.07		

SD = standard deviation.

### 3.2. Comparison

Means of posttreatment testosterone rise were compared between the 2 groups (Table 3). The posttreatment free testosterone rise in non-metformin group (M = 2.63, SD = 1.07) was significantly higher(mean difference = 0.80, 95% CI) than metformin group(M = 1.82, SD = 0.99, t = 4.76, *P* < .001). So, the table demonstrates significantly lower free testosterone rise after 30 days of treatment with metformin in contrast to the same duration treatment with other agents than metformin.

Bivariate Pearson’s product-moment correlation coefficient(r) was applied to see the correlation between Metformin dose and free testosterone level. Interestingly, a statistically significant positive correlation was found (Table 4). One-way ANOVA confirmed a significant difference across dose groups (*P* < .001).

**Table 4:** Correlation between metformin dose and free testosterone.

Metformin Dose (mg)	N	Mean ± SD
0	75	2.63 ± 1.07
750	3	1.61 ± 1.23
1000	24	1.86 ± 1.20
1500	32	1.76 ± 0.86
2000	16	1.95 ± 0.94
Total	150	2.23 ± 1.11

We searched for a correlation between age and free testosterone level. We found a statistically significant positive correlation among them (*P*-value <.001). That means older patients experience a greater rise in testosterone than younger patients (Table 5).

**Table 5:** ANOVA summary for relation between metformin dose and change in free testosterone.

Source	Sum of squares	df	Mean square	*F*	*P*-value
Between groups (combined)	24.720	4	6.180	5.687	<.001
Within groups	157.555	145	1.087	–	–
Total	182.274	149	–	–	–

## 4. Discussion

The negative effects of metformin on testosterone, as well as other components of sexual and reproductive functions, are now a matter of interest to many researchers.^[14,15]^

Several studies have shown subnormal levels of free testosterone in T2DM, increased BMI, and systemic inflammation separately.^[16–18]^ In diabetic male patients (whether type 1 or 2), hypogonadism is always a concern for patients as well as treating physicians, as testosterone reduction is correlated with decreased sexual function and poor muscle mass.

An increment in free testosterone level in proportion to good glycaemic control is expected. Studies correlating blood sugar and serum-free testosterone levels in T2DM patients are rare; several studies showed a positive correlation between insulin sensitivity and free and total testosterone levels.^[19]^ Initially, we assumed that metformin would suppress free testosterone levels similar to PCOS. However, after data analysis, it became evident that whether it was metformin or another antidiabetic drug, free testosterone was raised above the baseline in both cases. Improving glycaemic control may be the most important factor behind this increment in free testosterone levels.

When it came to comparing metformin and other drugs regarding raising free testosterone, the non-metformin group was statistically superior in our study. We observed that testosterone levels with treatment options other than metformin were significantly higher than in metformin-treated T2DM patients.

However, the correlation between the dose of metformin and its effect on testosterone was not statistically significant.

A study in China by Hu et al was conducted to compare the effect of insulin therapy and metformin on testosterone levels in T2DM patients. Their sample size was smaller (n = 70), drug-naïve newly diagnosed diabetic patients.^[8]^ Firstly, all patients were treated with an insulin pump to achieve normoglycaemia. Then, they were randomized between the metformin and insulin groups; a comparison was made between metformin and insulin. Metformin was found to lower testosterone.

Another interesting study was conducted by Ozata M et al among obese patients.^[7]^ They aimed to observe the effect of a combined low-calorie diet and metformin on serum testosterone levels and leptin levels. Patients were divided into 2 groups: diabetic and nondiabetic. They found that the levels of both total and free testosterone were significantly decreased with a low-calorie diet and metformin.

Another study used metformin, sitagliptin, and glibenclamide singly, in dual combinations, and all 3 together. They found that the combination of metformin and sitagliptin significantly and negatively affected sperm count, sperm quality, and testosterone levels in male rats.^[20]^

In the above-mentioned studies, authors found that metformin lowered testosterone levels. However, we found that metformin increases testosterone from baseline. We excluded patients with known hypogonadism, on antiandrogenic drugs, and also patients on statins. We didn’t see such exclusion criteria in their studies.

When we see that metformin is increasing testosterone levels, we have to consider the effect of improved glycemia also. In the above-mentioned study conducted by Y. Hu et al., we see that improved glycemic control is associated with improved free testosterone.^[8]^ Conversely another study showed that metformin, although it improves glycemic control, depressed testosterone level.^[21]^ So, evidence of the relation between glycaemic control and testosterone is mixed. Still, it may be an important confounding factor in our results.

The correlation between free testosterone level and insulin resistance is not clear. One study showed that low testosterone was associated with insulin resistance. But longitudinal observation didn’t demonstrate any effect of improving insulin resistance on testosterone levels.^[22]^ Authors concluded that it needs further studies to explore this correlation.

We found a positive correlation between metformin dose and free testosterone rise. The observed dose–response relationship indicates that the dose of metformin affects our results. Future studies with stratified dosing arms are warranted to confirm this relationship.

Because age is known to influence testosterone levels, we examined whether age differences might have explained our findings. However, the average ages of the 2 groups were virtually identical, and there was no statistically significant difference between them. This indicates that the groups were age-matched. So, when we searched for a correlation between age and rise in free testosterone, we found a positive correlation (*R* = 0.273, *P* = .001). This suggests that part of the difference observed between groups may be influenced by age (Table 6).

**Table 6:** Correlation between age and change in free testosterone.

Variable	Pearson’s r	*P*-value
Age in years vs change in free testosterone	.0273	.001

Here, it is worth mentioning that although some studies showed metformin to have some positive effects on androgenic parameters, those studies didn’t search for the relation between metformin and testosterone.^[23–26]^

However, many issues should still be explored, the effects of every single antidiabetic drug separately.

Considering other studies and ours, at least, we can say that antidiabetic medications (other than metformin) have a more positive effect on serum-free testosterone than metformin in male diabetic patients.

When we talk about testosterone levels, obviously, questions arise about sexual activity, fertility, and interrelationships among them. However, we observed only the effect of metformin on testosterone. We didn’t search for baseline sexual functional status, fertility status, or any other androgenic parameters or their relationships with metformin. We hereby recommend conducting further large-scale studies on the same issue and more studies to explore the relationship between testosterone levels and sexual activities in Bangladeshi people. There are very few studies on this issue worldwide.^[27–30]^

## 5. Limitations

A single measurement of free testosterone is not sufficient for testosterone-related decision-making. Drugs other than Metformin are diverse. Each different group of drugs has its merit. Consideration of individual drugs would be more effective.

Although the groups were age-matched at baseline, we found a significant positive correlation between age and the change in testosterone, demonstrating that older patients experienced a greater increase. Age may therefore be an independent predictor of testosterone change.

We did not collect data on other demographic and lifestyle factors such as exercise, smoking, alcohol consumption, sleep quality, stress, diet, and body mass index/waist–hip ratio, all of which are known to influence testosterone levels. These issues could have contributed to residual confounding. We suggest undertaking further studies, taking into consideration each of these factors.

Finally, the relatively short follow-up period of 30 days is not enough to determine the testosterone level. These factors should be considered in future studies with larger sample sizes and more comprehensive data collection.

## 6. Conclusion

Our study proves that metformin is inferior in raising free testosterone levels in contrast to other antidiabetic medications in male T2DM patients. This is an important issue for future researchers. As metformin has a central position in the T2DM management plan, and we see here it doesn’t suppress testosterone, so both physicians and patients can use it without worry, as testosterone is related to various androgenic parameters.

## Acknowledgments

We remember our colleagues with honor and gratefulness at Rajshahi Medical College, Bangladesh, and Rajshahi Diabetic Association General Hospital, Bangladesh.

## Author contributions

**Conceptualization:** Md Aminul Islam.

**Formal analysis:** Md Aminul Islam, Md Azizul Haque, Md Siddiqur Rahman.

**Methodology:** Md Aminul Islam, Abdul Mumit Sarkar, Md Siddiqur Rahman.

**Supervision:** Md Aminul Islam, Al Muksit Mohammad Taufiqur Rahman.

**Writing – original draft:** Md Aminul Islam.

**Writing – review & editing:** Md Aminul Islam, Shamrose Begum.
